# Patient‐reported outcomes in childhood head and neck rhabdomyosarcoma survivors and their relation to physician‐graded adverse events—A multicenter study using the FACE‐Q Craniofacial module

**DOI:** 10.1002/cam4.5252

**Published:** 2022-10-07

**Authors:** Michèle Morfouace, Marinka L. F. Hol, Reineke A. Schoot, Olga Slater, Daniel J. Indelicato, Frédéric Kolb, Ludwig E. Smeele, Johannes H. M. Merks, Charlene Rae, Heleen Maurice‐Stam, Anne F. Klassen, Martha A. Grootenhuis

**Affiliations:** ^1^ Department of Pediatric Oncology Princess Máxima Center for Pediatric Oncology Utrecht The Netherlands; ^2^ Department of Pediatric Surgery Princess Maxima Center Princess Máxima Center for Pediatric Oncology Utrecht The Netherlands; ^3^ Department of Otolaryngology and Head and Neck Cancer University of Utrecht Utrecht The Netherlands; ^4^ Department of Pediatric Oncology Great Ormond Street Hospital London UK; ^5^ Department of Radiotherapy University of Florida Proton Therapy Institute Jacksonville Florida USA; ^6^ Department of Plastic Surgery Institute Gustave Roussy Paris France; ^7^ Department of Pediatrics McMaster University Hamilton Canada

**Keywords:** cancer survivors, late adverse effects, patient‐reported outcome measures, quality of life, rhabdomyosarcoma, survivorship

## Abstract

**Introduction:**

Adverse events (AE) of treatment are prevalent and diverse in head and neck rhabdomyosarcoma (HNRMS) survivors. These AEs are often reported by physicians; however, patients' perceptions of specific AE are not well known. In this study, we explored patient‐reported outcomes measuring appearance, health‐related quality of life (HRQOL), and facial function in HNRMS survivors. Second, we assess the relationship between physician grading of AE and patient reporting.

**Materials and Methods:**

Survivors of pediatric HNRMS, diagnosed between 1993 and 2017, who were at least 2 years after completing treatment were invited to an outpatient clinic as part of a multicenter cross‐sectional cohort study. At the outpatient clinics, survivors aged ≥8 years filled out the FACE‐Q Craniofacial module; a patient‐reported outcome instrument measuring issues specific to patients with facial differences. AE were systematically assessed by a multidisciplinary team based on the Common Terminology Criteria of Adverse Events system.

**Results:**

Seventy‐seven survivors with a median age of 16 years (range 8–43) and median follow‐up of 10 years (range 2–42) completed the questionnaire and were screened for AEs. Patient‐reported outcomes varied widely between survivors. Many survivors reported negative consequences: 82% on appearance items, 81% on HRQOL items, and 38% on facial function items. There was a weak correlation between physician‐scored AEs and the majority of patient‐reported outcomes specific for those AEs.

**Conclusions:**

Physician‐graded AEs are not sufficient to provide tailored care for HNMRS survivors. Findings from this study highlight the importance of incorporating patient‐reported outcome measures in survivorship follow‐up.

## Abstract 2

Physician graded adverse effects are not sufficient to provide tailored care for head and neck cancer survivors. Patient reported outcome scores for appearance, health related quality of life and facial function was reported negatively by many survivors. This study highlights the importance of incorporating patient reported outcome measures in follow‐up clinic.

## 1 INTRODUCTION

Rhabdomyosarcoma (RMS) accounts for around 4% of all childhood cancers and originates in the head and neck (HN) area in 40% of patients.^1^ Survival has increased significantly since the use of multimodality therapy, including local treatment with radiotherapy, and in some cases, added surgery. However, both radiotherapy and surgery damage healthy tissues. This damage can cause a wide range of adverse events (AEs) in survivors, including visible facial differences, ocular impairment, hearing impairment, speech abnormalities, and endocrinopathies.^2^, ^3^, ^4^, ^5^, ^6^, ^7^ With more patients becoming long‐term survivors, AEs are an important topic. The Common Terminology Criteria of Adverse Events (CTCAE)^8^ is a clinical grading system used to report AEs.^9^ However, the relation between the grade of AEs and the patients' perception of those AEs is not consistent in adult studies^10^, ^11^, ^12^ and is not well described for children and adolescents. A better understanding of the patients' perception could improve the quality of care for survivors.

Our group^13^ has previously reported on the psychosocial well‐being of a partially overlapping cohort of 65 childhood HNRMS survivors. That study showed that health related quality of life (HRQOL) of survivors was comparable to general population norms on most psychosocial domains. However, survivors reported disease‐specific issues such as negative self‐image and lack of satisfaction with appearance. To further characterize these issues, condition‐specific patient‐reported outcome (PRO) instruments can be used. It was previously shown that the majority of available PROs for children and youth with craniofacial conditions contain limited appearance and facial function items and lack content validity.^14^ To address this limitation, the FACE‐Q Craniofacial module was developed.^15^ This PRO instrument is composed of a comprehensive set of independently functioning scales that are applicable to a wide range of conditions associated with facial differences, including childhood cancer. The scales measure outcomes related to appearance, HRQOL, and facial function.

The aim of the present study was to explore specific PROs for appearance, HRQOL, and facial function within a cohort of pediatric HNRMS survivors, using relevant scales from the FACE‐Q Craniofacial module. We explored differences between survivors in terms of gender, age at diagnosis, attained age, follow‐up period, tumor site, laterality, and local treatment strategy. Second, we assessed relationships between physicians' grading of AEs and specific PROs.

## 2 METHODS

### 2.1 Setting

Survivors were recruited from five international centers: Great Ormond Street Hospital, London, United Kingdom; University of Florida Health Proton Therapy Institute, Florida, United States; Institute Gustave Roussy, Paris, France; Emma Childrens' Hospital, Amsterdam, which later transferred all pediatric care to the Princess Máxima Center for pediatric oncology, Utrecht, The Netherlands. Survivors of pediatric (0–18 years) HNRMS, diagnosed between 1993 and 2017 who were ≥2 years after completion of treatment were eligible. All survivors were treated with multiagent chemotherapy and local treatment.^1^, ^16^, ^17^ Four local treatment strategies were available during the period studied: definitive external beam radiation with photons (RT); definitive external beam radiation with protons (PT); microscopically (R0) radical surgery combined with RT or PT (the Paris‐method); macroscopic radical surgery combined with brachytherapy (AMORE).^18^ Data on AEs were collected during standardized multidisciplinary outpatient clinics held between January 2017 and December 2019. Survivors aged ≥8 years were also invited to complete the FACE‐Q Craniofacial scales before clinic; they were sent by mail or given when entering the outpatient clinic. Oral or written informed consent was obtained based on national and local standards. In the United Kingdom and United States, this study was approved by the national and local ethics committee and written consent was obtained from all participants. In the Netherlands and in France, this study was exempted from ethical approval as the study fell under regular healthcare practices.

### 2.2 Patient‐reported outcomes

We used 11 of the FACE‐Q Craniofacial module^15^ scales that were developed as part of the CLEFT‐Q (15) and field‐tested in a large sample of noncleft craniofacial patients.^19^, ^20^ Each scale containing 7–12 items, answered on a 1–4 Likert scale. This PRO instrument assesses concepts from three different domains: appearance (of face, nose, teeth, lips, and jaw), HRQOL (psychological, social, and school function and speech distress), and facial function (speech function and eating & drinking). The appearance scales ask how much the respondent like their current appearance. The HRQOL and facial function scales ask respondents how often or how much a set of statements applied to them in the previous week. Participants completed only relevant scales (e.g., jaws, for participants aged ≥12 year; school, for participants aged ≤18 year and attending school). The eating & drinking scale was only used as an item checklist.^21^ For all other scales, the sum score of items was available as a Rasch transformed score^22^ from 0 to 100. Lower scores reflect worse outcome. Internal consistency of scales was good,^23^ with Cronbach's alpha between 0.83 and 0.97 in our cohort. If missing data comprised <50% of the scale's items, the mean of the completed items for a scale was used, otherwise a score was excluded for that survivor.

### 2.3 AE assessment

A predefined list of AEs were graded according to CTCAE 4.0^1^, was added to Supplemental Data A. We assessed musculoskeletal deformity, short stature (<‐2SD), speech abnormalities, oral malfunction (trismus, xerostomia, taste alterations), hearing impairment, ocular impairment, and facial nerve paresis. AEs were dichotomized into </≥ grade 2 to reflect the absence/presence of a clinically relevant problem (i.e., being symptomatic, requiring alterations in activities of daily living, and/or the need for an intervention or medication) (Supplemental Data A).

### 2.4 Statistical analysis

Data were analyzed with SPSS version 26.0. To explore PRO scores, mean and standard deviations (±SD) were calculated for the scales, for the whole cohort and for subgroups. Subgroups were based on: gender, age at diagnosis, attained age, follow‐up period, tumor site, laterality, and treatment strategy. Differences between subgroups were tested with a one‐way ANOVA and/or independent sample *t*‐test. Differences between appearance scale scores within survivors were tested with a dependent *t*‐test. Effect sizes (Cohen *d*) were calculated and considered as: 0.2 small, 0.5 medium, and ≥0.8 large.^24^ Correlations between scale scores were calculated with Pearson correlation coefficient (r) and considered as: 0.1 weak, 0.3 medium, and ≥0.5 strong.^24^

To get more detailed insight, item level analyses were explored. We calculated the percentage of survivors that reported negatively for items on the appearance scales (i.e., “not at all,” “a little bit”), HRQOL scales (i.e., “never,” “sometimes”) and speech distress, speech function, and eating & drinking scales (i.e., “always,” “often”).

To assess the relation between grading of AEs and PRO scores, we compared the mean scale scores of the survivors with a clinically relevant AE to that of survivors without a clinically relevant AE, using independent sample *t*‐test and Cohen's *d*. For the psychological and social scales, the relation with every AE was assessed. In addition, appropriate scales were examined per AE. The relation of the number of different AEs with the psychological and social scale scores was examined with Spearman rho test.

## 3 RESULTS

### 3.1 Survivors

Ninety‐five survivors aged ≥8 years attended the clinics. Seventy‐seven (81%) completed the questionnaire. The 18 nonparticipants were more often treated with the Paris‐method compared to the participants (*p* = 0.004) (Table S1). Table 1 presents the survivor's demographic and clinical characteristics.

**TABLE 1:** Characteristics of the participants (total *N* = 77)

Gender, male *N* (%)	43 (56)
Age at diagnosis, y *Median (min–max)*	6 (0–16)
Age at clinic, y *Median (min–max)*	16 (8–43)
Follow‐up duration, y *Median (min–max)*	10 (2–42)
Site, *N* (%)	
PM	45 (58)
NPM	12 (16)
orbit	20 (26)
Country of residence, *N* (%)	
United Kingdom	31 (40)
United States	6 (8)
France	8 (10)
The Netherlands	32 (42)
Local treatment received, *N* (%)	
RT	32 (42)
Protons	22 (29)
AMORE	18 (23)
Paris‐method	5 (6)

Abbreviations: AMORE, ablative surgery MOuld placement and Reconstruction; NPM, head and neck non‐parameningeal’; PM, parameningeal; RT, external beam radiotherapy with photons; Y, years.

For 76 of the 77 participants (99%), CTCAE grading was available. Sixty‐three (82%) had ≥1 AEs, 29 (38%) ≥2 AEs, with a maximum of 5 AEs in 2 (3%) survivors (Figure S1).

### 3.2 Exploring patient‐reported outcomes

The face, psychological, school, and social scales are presented in Table 2. Table S2 shows the scales concerning specific aspects of the face (nose, teeth, lips, jaw), and the speech distress and speech function scales. The prevalence of negative reporting at item level is presented in Table 3.

**TABLE 2:** Mean scale scores^a^ (± standard deviations [SD]) on appearance of the face, psychological function, school function, and social function

		Domain							
		Appearance		HRQOL					
		Face (*N* = 77)		Psychological (*N* = 76)		School^b^ (*N* = 41)		Social (*N* = 76)	
	*N* (%)	Mean	SD	Mean	SD	Mean	SD	Mean	SD
All	77	54.0	16.1	64.9	17.8	69.2	17.1	70.1	16.3
		**Min**	**Max**	**Min**	**Max**	**Min**	**Max**	**Min**	**Max**
		7	100	15	100	42	100	32	100
	***N* (%)**	**Mean**	**SD**	**Mean**	**SD**	**Mean**	**SD**	**Mean**	**SD**
Gender									
Male	43 (56)	52.7	16.8	63.6	18.9	67.1	19.2	69.3	16.1
Female	34 (44)	56.3	15.2	66.4	16.4	71.8	14.5	71.0	16.9
Age at diagnosis (median, range)	*6 (0–16)*								
0–5 years	43 (56)	55.5	14.4	66.7	15.3	70.4	17.5	69.8	14.9
6–9 years	22 (29)	52.5	14.7	61.7	20.5	68.5	16.0	70.9	18.6
≥10 years	11 (14)	52.9	25.3	65.0	22.1	65.4	20.6	70.4	18.6
Attained age (median, range)	*16 (8–43)*								
8–12 years	21 (27)	**59.5** ^c^	16.6	**75.2** ^**d**^	16.0	70.9	16.2	72.6	17.1
13–17 years	23 (30)	49.2	15.9	61.7	20.3	67.7	18.3	66.5	19.7
≥18 years	33 (43)	54.6	15.3	60.8	14.5	‐	‐	70.8	13.2
Follow‐up duration (median, range)	*10 (2–42)*								
2–5 years	19 (25)	52.4	22.1	67.1	22.9	70.7	16.1	70.1	17.9
6–9 years	21 (27)	58.9	14.9	71.9	16.9	68.6	18.7	71.1	20.6
≥10 years	37 (48)	52.7	12.8	**59.8** ^e^	13.9	67.3	17.3	69.4	12.8
Site^f^									
PM	47 (61)	53.4	16.4	64.4	17.9	69.5	17.8	70.6	16.5
NPM	11 (14)	54.9	15.8	62.6	10.5	62.2	11.8	65.5	15.2
Orbit	19 (25)	56.4	16.3	67.6	21.1	71.8	18.0	71.3	17.0
Laterality									
Lateral	63 (82)	53.8	17.2	65.3	19.0	69.2	17.1	69.8	16.7
Midline	12 (16)	56.7	10.3	62.5	12.0	69.7	19.1	71.1	16.0
Local treatment									
RT	32 (42)	55.1	16.9	61.0	18.4	65.6	15.9	67.4	17.0
Proton	22 (29)	58.2	14.8	**73.2** ^g^	16.4	68.2	16.1	71.9	18.5
AMORE	18 (23)	52.4	13.3	65.0	13.2	77.2	22.8	72.9	13.8
Paris‐method	5 (6)	**38.8** ^h^	20.5	52.0	21.1	74.8	18.2	68.0	10.7

*Note*: In bold; statistically significant difference between groups.

^a^ Mean Rasch transformed scores on scale 0–100; higher scores reflecting better outcome.

^b^ Only fulfilled by survivors aged <18 years and attending school.

^c^ Survivors aged 8–12 years scored significantly higher compared to survivors aged 13–17 years (*d* 0.6, *p* = 0.041).

^d^ Survivors aged 8–12 years scored significant higher compared to survivors aged 13–17 years (*d* 0.7, *p* = 0.021) and survivors aged ≥18 years (*d* 1.0, *p* = 0.001).

^e^ Survivors with a follow‐up duration ≥10 years scored significantly lower compared to survivors with follow‐up duration 6–9 years (*d −*0.8, *p* = 0.005).

^f^ ‘PM’: parameningeal site, ‘NPM’: head and neck non parameningeal site, ‘orbit’: orbital site.

^g^ Survivors treated with proton scored significantly higher compared to survivors treated with Paris‐method (*d* 1.2, *p* = 0.020) or RT (*d* 0.7, *p* = 0.016).

^h^ Survivors treated according to the Paris‐method scores significantly lower compared to survivors treated with protons (*d* −1.2, *p* = 0.020).

**TABLE 3:** Percentage of survivors reporting negatively on the scale items of (A) appearance, that is, “not at all” or “a little bit” (B) psychological, social, and school, that is, “never” or “sometimes” (C) speech distress, speech function, and eating & drinking, that is, “always” or “often.” Items negatively reported by ≥20% of survivors in bold. Items negatively reported by ≥50% of survivors with*

A					
How much do you like…	Face	Nose	Teeth	Lips	Jaw
Sides match	60*	12	—	—	—
Photos	58*	13	—	15	**23**
Laugh	**49**	—	—	**24**	—
Up close	**48**	—	55*	15	—
Smile	**42**	16	**48**	**20**	**26**
From the side	**38**	**25**	**39**	—	**30**
Shape	**34**	16	—	15	**24**
Look your best	**26**	—	—	—	—
Ready to go out	**21**	—	—	—	—
Mirror	—	17	—	15	**26**
Size	—	13	**31**	13	**24**
Closed	—	—	—	16	**21**
Top and bottom meet	—	—	61*	—	—
Show when smile	—	—	51*	—	—
Straight	—	13	**44**	—	—
Close together	—	—	**39**	—	—
Full	—	—	—	15	—
Length	—	13	—	—	—
Middle part	—	16	—	—	—
Bottom	—	10	—	—	—
Tip	—	10	—	—	—

B					
Psychological	%	Social	%	School	%
Feel good	**47**	Same as others	**30**	Make friend	**31**
Feel great	**33**	Make friends	**30**	Join activities	**24**
Feel confident	**30**	People look	**29**	Happy	**22**
Happy with life	**26**	Confident out	**28**	Listen to me	**20**
Like self	**24**	Fit in	**24**	Safe	18
Believe in self	**24**	Being with others	16	Seeing friends	13
Proud of self	**22**	People listen	13	Nice to me	11
Feel happy	**22**	Treat the same	11	Teachers	11
Feel okay	**21**	Fun with friends	5	Feel accepted	11
Enjoy life	16	Friends accept	5	Liked	9

C					
Speech distress	%	Speech function	%	Eating & drinking	%
Not understood	**29**	Slowly	18	Slowly	**22**
Repeat	**26**	Read out loud	16	Trouble biting	18
Worry	16	Try hard	13	Hard to chew	18
Nervous	12	Concentrate	13	Gets stuck*^a^	18
Avoid	7	Repeat	12	Certain foods	14
Frustrated	7	Avoid words	10	Trouble straw	12
Embarrassed	4	Trouble words	10	Food falls out	11
Teased	5	On the phone	9	Small bites	9
Avoid going out	4	Family	9	Up my nose*^b^	3
New friends	4	Sentences	8		
		New people	8		
		Friends	7		

*Note*: —: Item not applicable in scale.

*Note*: ^a,b^Items only available in the Dutch and French version of FACE‐Q Craniofacial Module (at the time of our study). ^a^ *N* = 39; ^b^ *N* = 31.

### 3.3 Appearance

The distribution of scores on the face scale varied widely: range 7–100. The mean face score was significantly higher for survivors aged 8–12 years compared to survivors aged 13–17 years (*d* 0.6). The mean score on the lips scale was significantly higher for survivors aged 8–12 years compared to older survivors (13–17 years *d* 0.7; ≥18 years *d* 0.8). Mean lips and jaw scores were significantly higher for orbit site compared to PM site (*d ≥* 0.9). Mean face score was significantly lower for survivors treated according to the Paris‐method compared to survivors treated with protons (*d* − 1.2). Mean lips score was significantly lower for survivors treated according to the Paris‐method compared to survivors treated with protons (*d* − 1.3) or AMORE (*d* − 1.2).

Within survivors, scores on appearance of the lips, nose, and jaw were significantly higher compared to their face score (*d* 0.9, 0.8, 0.5, respectively).

Sixty‐three (82%) survivors reported negatively on ≥1 of the appearance‐scales items. Every item of the face, jaw, and teeth scales was reported on negatively by >20% of survivors. Sixty percent of survivors reported negatively on the item “…how well both sides of your face match.”

### 3.4 HRQOL

The mean psychological scale score was significantly higher for survivors aged 8–12 years compared to older survivors (13–17 years *d* 0.7; ≥18 years *d* 1.0). Survivors with ≥10 years follow‐up had lower mean psychological score compared to those with shorter follow‐up (6–9 years *d −* 0.8). The mean psychological score was significantly higher for survivors treated with protons compared to survivors treated with RT (*d* 0.7) or the Paris‐method (*d* 1.2).

Sixty‐two (81%) survivors reported negatively on ≥1 of the HRQOL‐scales items. Nearly half (47%) of all survivors reported negatively on the item “I feel good about how I look.”

### 3.5 Facial function

The mean speech function score was significantly higher for AMORE‐treated survivors compared to the survivors treated with RT (*d* 1.1), protons (*d* 0.9), or the Paris‐method (*d* 1.3). Eighteen percent of survivors reported that they need to speak slowly to be understood. Twenty‐nine (38%) survivors reported negatively on ≥1 of the speech function items. Twenty‐eight (36%) survivors reported negatively on ≥1 of the eating & drinking items.

Strong correlations (*r* ≥ 0.5) across the domains were seen for the: face and psychological scale; face and social scale; and speech function and speech distress scale (Table S3).

### 3.6 Relation between AEs and PROs

**FIGURE 1:**
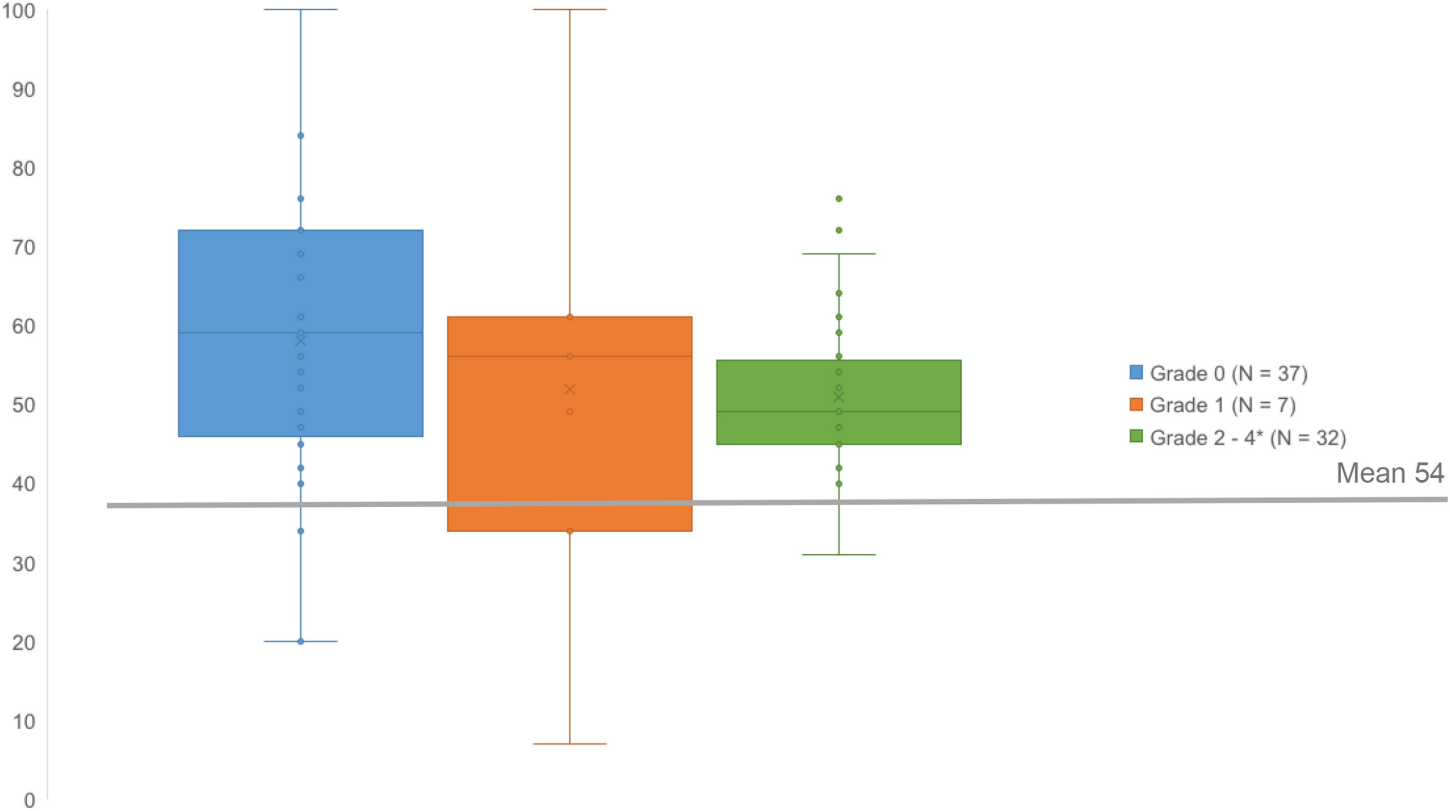
Face scale score per grade of musculoskeletal deformity (0–4). *grade 2 *N* = 9, grade 3 *N* = 21, grade 4 *N* = 2.

Both the highest and the lowest scores on the face scale were reported by the survivors with a grade 0 or 1 deformity (Figure 1). No differences were seen between survivors with or without a musculoskeletal deformity grade ≥2 on any of the tested scales (Table 4).

**TABLE 4:** Mean^a^ and standard deviation of the PRO scale scores for survivors without and with (*N* = X/X) a physician‐graded AE grade ≥2

	AE grade <2		AE grade ≥2			
Scale	Mean	SD	Mean	SD	*d* ^*b*^	p^c^
	Musculoskeletal deformity (*N* = 44/32)					
Psychological	67.0	18.8	62.2	16.5	−0.3	0.32
Social	71.9	17.7	67.8	14.5	−0.2	0.41
School	70.0	15.9	68.1^d^	19.4	−0.1	0.99
Face	57.1	19.1	51.0	10.2	−0.4	0.08
Nose	69.6	22.5	66.6	16.5	−0.1	0.57
Teeth	53.0	21.0	50.8	17.5	−0.1	0.67
Lips	76.0	23.4	67.2	19.7	−0.4	0.11
Jaw	65.7	24.1	59.6^e^	21.9	−0.3	0.21
	Short stature (*N* = 70/6)					
Psychological	65.0	18.4	62.4	9.2	−0.1	0.76
Social	69.9	16.4	70.9	19.2	0.1	0.91
	Speech abnormality (*N* = 57/11)					
Psychological	65.4	18.3	64.2	12.3	−0.1	0.83
Social	71.1	16.9	64.3	11.0	−0.3	0.20
School	71.2	17.0	58.7^d^	7.7	**−0.8**	0.09
Speech distress	77.3	17.1	65.9	15.5	−0.7	0.04*
Speech function	78.7	17.3	55.6	16.3	**−1.3**	0.00*
	Oral malfunction (*N* = 60/8)					
Psychological	65.5	17.3	62.9	19.4	−0.1	0.69
Social	70.3	15.3	67.4	21.4	−0.2	0.63
Speech distress	75.9	17.6	72.6	15.3	−0.2	0.62
Speech function	75.4	19.5	72.3	15.5	−0.2	0.66
Teeth	51.0	17.8	56.3	19.3	0.3	0.44
Lips	72.4	22.2	65.4	23.0	−0.3	0.41
Jaw	63.5	22.5	44.0^e^	15.8	**−0.9**	0.03*
	Hearing impairment (*N* = 59/13)					
Psychological	65.9	17.7	56.7	18.0	−0.5	0.26
Social	70.5	16.3	68.8	14.6	−0.1	0.73
School	70.4	16.8	67.7^d^	17.9	−0.2	0.71
Speech distress	76.1	17.1	72.8	17.8	−0.2	0.53
Speech function	76.2	18.6	70.2	20.5	−0.3	0.30
	Ocular impairment (*N* = 30/36)					
Psychological	66.8	17.0	65.3	17.8	−0.1	0.99
Social	71.3	15.2	70.0	18.2	−0.1	0.66
School	69.6	17.2	72.4^d^	19.2	0.2	0.69
	Facial nerve paresis (*N* = 64/6)					
Psychological	66.0	17.7	58.5	23.9	−0.4	0.34
Social	70.2	16.5	72.8	14.5	0.2	0.71
Face	56.0	16.3	42.2	18.0	**−0.8**	0.05*
Lips	73.9	22.0	59.5	25.3	−0.6	0.14
Speech function	73.7	19.4	83.2	15.9	0.5	0.25

^a^ Mean Rasch transformed scores on scale 0–100; higher scores reflecting better outcome.

^b^ Effect sizes, large (≥0.8) effect sizes are presented in bold.

^c^ Statistical significance of the difference in means, difference at the *p* ≤ 0.05 level shown with an asterix.

^d^ School scale only filled out by children aged ≤18 and attending school: musculoskeletal deformity *N* = 16, speech abnormality *N* = 6, hearing impairment *N* = 7, ocular problem *N* = 21 in the category with an AE grade ≥2. Results for short stature are not presented because of very small number of survivors with the AE present (*N* = 2).

^e^ Jaw scale only filled out by participants aged ≥12 years: musculoskeletal deformity *N* = 27, oral malfunction *N* = 7 in the category with an AE grade ≥2.

Large (*d ≥* 0.8) differences in some PRO scale scores between survivors with and without a clinically relevant AE were seen for: speech abnormality, oral malfunction, and facial nerve paresis (Table 4), with lower scores for the survivors with the AE present.

The number of different AEs was nonsignificantly, weakly associated with the mean psychological and social scores (*r* − 0.106 and − 0.129. respectively) (Figure S2).

## 4 DISCUSSION

The PROs scores for appearance, HRQOL, and facial function varied widely in this cohort of HNRMS survivors. Many survivors reported negative consequences: 82% on appearance items, 81% on HRQOL items, and 38% on facial function items. PRO scores across the three domains were associated with each other. The correlation between the presence of a clinically relevant AE as graded by physicians and PROs was weak for the majority of the tested PROs, and strong for only a few.

Our group published previously on a partially overlapping cohort,^13^ and showed HNRMS survivors experienced negative disease‐specific issues. In the current study, we further characterized these issues by using a questionnaire designed to measure facial appearance and function in addition to HRQOL. The FACE‐Q Craniofacial module is the first PRO instrument designed for children and young adults to appraise their appearance rather measure appearance distress.

In general, the scores of survivors with clinically relevant AEs did not differ significantly on appearance, HRQOL, and facial function scales compared to those of survivors without these AEs. We only observed lower scores on a few specific scales for survivors with a speech abnormality, oral malfunction, and facial nerve paresis compared to the survivors without these problems. These findings suggest AE categorization by physicians does not account for patient perspective. Similar findings have also been observed in the adults cancer literature, with multiple studies reporting weak to moderate correlation between CTCAE grading and associated PROs.^12^ These findings have led to the development of a patient language version of the CTCAE (CTCAE‐PRO),^25^ to complement the CTCAE and incorporate patient reporting of symptoms more systematically into research and decision making. The described weak correlation between physician reporting and PROs provides further support to the theories that claim factors other than the presence of a chronic condition affect the consequences of the condition on an individuals' psychosocial well‐being.^26^, ^27^, ^28^, ^29^ Overall, HRQOL is lower in groups of people with a visible facial difference compared to groups without such a difference, but large individual variations exist.^30^, ^31^, ^32^, ^33^ These variations may be attributable to multiple psychological and social factors (i.e., personality, coping strategies, social support)^28^, ^34^, ^35^, ^36^ which warrant further investigation.

In our study, survivors with younger age (8–12 years) and shorter follow‐up time (<10 years) scored significantly higher on appearance and HRQOL than older survivors and longer follow‐up time. Similar findings were observed in a large international cohort of patients with cleft lip/palate, assessed with partly overlapping scales from the CLEFT‐Q.^21^ This age and time effect might be explained by the importance of appearance during different developmental stages.^29^ In addition, in HNRMS survivors, facial deformity may aggravate over time with the growth of the facial bones. Some differences in scoring on appearance, HRQOL, and facial function scales were seen between survivors treated with different local treatment strategies. These differences should be interpreted cautiously because of differences in patient characteristics (Data Table S4), especially in terms of tumor site, attained age, and follow‐up time. Besides that, the Paris‐method is used in a specific subgroup of PM‐site tumors with a worse prognosis and is aimed at improving survival. This might lead to a different definition of acceptable toxicity. Additionally, local treatment strategy is partly dependent on the country of treatment. Differences in scoring might reflect underlying differences in country‐specific HRQOL.

Within our cohort. we did not find differences in subgroups based on gender, age at diagnosis, and laterality. Previous studies on HRQOL in childhood cancer survivors have described more negative scoring on emotional health for females compared to males,^37^, ^38^ and on worry and social function for patients with older age at diagnosis compared to younger age at diagnosis.^38^ This difference with our results might be explained by the specific (instead of generic) HRQOL items included in the current study that do not address these general HRQOL domains.

### 4.1 Strengths and limitations

We present an international cohort of HNRMS survivors with long follow‐up. Our results on specific aspects of appearance, HRQOL, and facial function give a detailed description of the issues HNRMS survivors' experience.

An important limitation of the study is inherent to the population under investigation: patient numbers are small and cohorts heterogeneous. Therefore, the results are mainly exploratory and the analyses have limited power.

To date, normative values were not available for the FACE‐Q Craniofacial module, which impairs interpretation of our results in reference to the general population. Ideally, our data would be compared to a general population control group or a childhood cancer survivor group in whom cancer treatment has not affected the head and neck area. The larger portion of our currently described cohort was used for a validation study which is in preparation for publication^39^ and reference values are expected to follow from this. However, given the intended use to improve care for individual survivors, we do believe that the use of the FACE‐Q Craniofacial module without existing normative values adds value in the clinical setting to address unmet medical needs by giving a clear insight in the specific problems the individual survivor experiences. Once reference values become available, future research can use these to evaluate whether interventions (both psychological and/or surgical) initiated based on problems identified via de FACE‐Q Craniofacial module helped to improve individual patients' outcomes. Furthermore, for the individual survivor, changes in scoring over time can be objectified.

Important to take into account are the differences in patient and treatment characteristics between the participants and nonparticipants. The nonparticipants were more often treated with the Paris‐method and had PM site tumors. The combination of these factors was unsurprising since the Paris‐method is developed for PM site tumors. This method includes extensive surgical tumor resection and thereby introduces a risk of significant facial deformation. Because of this, a proportion of the objectively more severely affected children have not been included in the current study. However, only a minority of all international HNRMS patients are treated according to this method. The reasons for not participating was not documented as this is not a permitted question by most ethical boards.

### 4.2 Clinical implications

Many survivors reported negatively on appearance, HRQOL, and facial function items. Relying on the physician‐graded AEs is not enough to provide tailored care to the survivors because of the weak correlation between AEs and the majority of PRO scores. We recommend health care professionals to pay attention to issues on all three domains in every HNRMS survivor. The FACE‐Q Craniofacial module can be used to obtain this goal. Training to help physicians use PROs in clinical care and how to discuss these with their patients is recommended in order to incorporate the patients' perspective next to objective measures of AEs.^40^ The systematic use of questionnaires can be facilitated by the use of electronic portals such as the Dutch “Kwaliteit van Leven In Kaart” (KLIK) PROM portal.^41^ In this portal, patients are asked to complete online PROs at home before a consultation. Scores are then converted into an individual electronic profile and discussed during the consultation. The use of PROs in clinical practice has been shown beneficial as it resulted in increased discussion of patient outcomes, enhanced patient–clinician communication, higher patient satisfaction, better HRQOL, and improved treatment outcomes.^42^, ^43^ Furthermore, children should be provided if possible with psychosocial interventions to empower them in coping with the consequences of their disease^44^ We would recommend to add PRO assessment to outpatient clinic visits but no more than once a year, given the possible change in scoring over time dependent on the survivors age and development of the face and consequently facial function. Currently, in the Netherlands, all head and neck sarcoma survivors are invited to a multidisciplinary follow‐up clinic every 2 years, at least until the age of 18 years and we will invite them to fill out the questionnaire during each visit.

## 5 CONCLUSION

PRO scores for appearance, HRQOL, and facial function varied widely between HNRMS survivors, though many survivors reported negative consequences in all three domains. The presence of clinically relevant AEs as graded by physicians was weakly correlated with the majority of disease specific PRO scores. We therefore advise a systematic assessment of potential concerns from the patient perspective, such as by use of the FACE‐Q Craniofacial module, in the care for every individual HNRMS survivor.

## AUTHOR CONTRIBUTIONS

Marinka L.F. Hol contributed to conception and design; contributed to acquisition, analysis, and interpretation; drafted the manuscript gave final approval; and agreed to be accountable for all aspects. Michèle Morfouace contributed to analysis and interpretation; drafted the manuscript, and agreed to be accountable for all aspects. Reineke A. Schoot contributed to conception and design; contributed to acquisition, analysis, and interpretation; critically revised the manuscript; gave final approval; and agreed to be accountable for all aspects. Olga Slater contributed to acquisition, analysis, and interpretation; critically revised the manuscript; gave final approval; and agreed to be accountable for all aspects. Daniel J. Indelicato contributed to acquisition, analysis, and interpretation; critically revised the manuscript; gave final approval; and agreed to be accountable for all aspects. Frédéric Kolb contributed to acquisition, analysis, and interpretation; critically revised the manuscript; gave final approval; and agreed to be accountable for all aspects. Prof. Ludwig E. Smeele contributed to conception and design; contributed to acquisition, analysis, and interpretation; critically revised the manuscript; gave final approval; and agreed to be accountable for all aspects. Johannes H.M. Merks contributed to conception and design; critically revised the manuscript; gave final approval; and agreed to be accountable for all aspects. Charlene Rae contributed to conception and design; contributed to acquisition, analysis, and interpretation; critically revised the manuscript; gave final approval; and agreed to be accountable for all aspects. Heleen Maurice‐Stam contributed to acquisition, analysis, and interpretation; critically revised the manuscript; gave final approval; and agreed to be accountable for all aspects. Anne F. Klassen contributed to conception and design; contributed to acquisition, analysis, and interpretation; critically revised the manuscript; gave final approval; and agreed to be accountable for all aspects. Martha A. Grootenhuis contributed to acquisition, analysis, and interpretation; critically revised the manuscript; gave final approval; and agreed to be accountable for all aspects.

## FUNDING INFORMATION

This research was supported by the Dutch Children Cancer free foundation (KIKA) under grant number 297.

## CONFLICT OF INTEREST

The authors report no conflict of interest.

## Supporting information


Table S1
Figure S1
Table S2
Table S3
Figure S2
Table S4

Appendix S1

## Data Availability

Data are available for review on request.
