# Supplementary material for: Patient‐reported outcomes in childhood head and neck rhabdomyosarcoma survivors and their relation to physician‐graded adverse events—A multicenter study using the FACE‐Q Craniofacial module
Source: Cancer Med. 2022 Oct 7;12(4):4739–50. doi: 10.1002/cam4.5252 (PMC9972026; doi:10.1002/cam4.5252)
Supplement: Supplementary file 1 — Table S1 Figure S1 Table S2 Table S3 Figure S2 Table S4 [file CAM4-12-4739-s001.docx]

Supplemental data

|  | **Participants**  **N = 77** | **Non-participants**  **N = 18** | **p** |
| --- | --- | --- | --- |
| **Gender, N (%)** |  |  | 0.794 |
| Male | 43 (56) | 11 (61) |  |
| Female | 34 (44) | 7 (39) |  |
| **Age at diagnosis, years** |  |  |  |
| *Median (range)* | *6 (0 – 16)* | 6 (1 – 15) | 0.779 |
| 0 - 5 | 43 (56) | 10 (56) |  |
| 6 - 9 | 22 (29) | 5 (28) |  |
| ≥ 10 | 11 (14) | 3 (17) |  |
| **Attained age, years** |  |  |  |
| *Median (range)* | *16 (8 – 39)* | 14 (8 – 34) | 0.568 |
| 8 - 12 | 21 (27) | 7 (39) |  |
| 13 - 17 | 23 (30) | 4 (22) |  |
| ≥ 18 | 33 (43) | 7 (39) |  |
| **Follow-up duration, years** |  |  |  |
| *Median (range)* | *10 (2 – 30)* | 8 (4 – 29) | 0.654 |
| 2 - 5 | 19 (25) | 3 (17) |  |
| 6 - 9 | 21 (27) | 8 (44) |  |
| ≥ 10 | 37 (48) | 7 (39) |  |
| **Local treatment, N (%)** |  |  | **0.004** |
| RT | 32 (42) | 5 (28) |  |
| protons | 22 (29) | 5 (28) |  |
| AMORE | 18 (23) | 1 (6) |  |
| Paris-method | 5 (6) | 7 (39)^a^ |  |
| **Site, N (%)** |  |  | 0.054 |
| PM | 47 (61) | 16 (89)^b^ |  |
| NPM | 11 (14) | 0 |  |
| orbit | 19 (25) | 2 (11) |  |
| **Side, N (%)** |  |  | 0.114 |
| Lateral | 63 (82) | 18 (100) |  |
| Midline | 12 (16) | 0 |  |
| **Adverse Events grade ≥2** |  |  |  |
| Musculoskeletal deformity | 42% | 73% |  |
| Speech abnormality | 16% | 20% |  |
| Oral malfunction | 11% | 30% |  |
| Hearing impairment | 18% | 11% |  |
| Ocular problems | 55% | 50% |  |
| Facial palsy | 9% | 29% |  |
| Short stature | 16% | 30% |  |

Supplemental table 1:

Characteristics for participants and non-participants

‘y’: years

‘PM’: parameningeal site, ‘NPM’: head and neck non parameningeal site, ‘orbit’: orbital site

RT: external beam radiotherapy with photons

AMORE: Ablative surgery MOulage brachytherapy and REconstruction

^a^ statistically significant differences according to Fisher exact: more Paris-method in the non-responders compared to the responders group (p = 0.001)

Supplemental figure 1: Venn diagram showing the overlap between the different adverse events within survivors


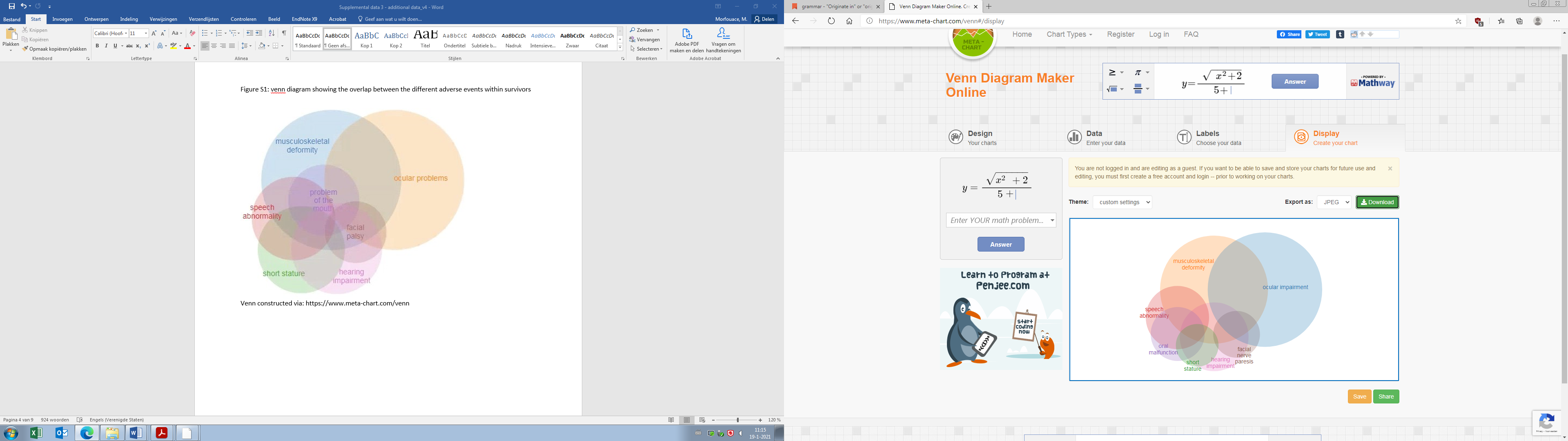


Venn constructed via: https://www.meta-chart.com/venn

Supplemental table 2: mean scale scores^a^ on appearance of the face, psychological function, school function and social function: exploring differences within the cohort, based on patient and tumor characteristics

|  | ***Domain*** | **Appearance** | | | | | | | | **HRQOL** | | **function** | |
| --- | --- | --- | --- | --- | --- | --- | --- | --- | --- | --- | --- | --- | --- |
|  | ***Scale*** | **Nose** | | **Teeth** | | **Lips** | | **Jaw^b^** | | **Speech distress** | | **Speech function** | |
|  | **N (%)** | **Mean** | **SD** | **Mean** | **SD** | **Mean** | **SD** | **Mean** | **SD** | **Mean** | **SD** | **Mean** | **SD** |
| **All** | 77 | 68.2 | 20.0 | 52.2 | 19.4 | 72.1 | 22.1 | 62.8 | 22.9 | 75.7 | 17.4 | 75.2 | 19.0 |
|  |  | **Min** | **Max** | **Min** | **Max** | **Min** | **Max** | **Min** | **Max** | **Min** | **Max** | **Min** | **Max** |
|  |  | 0 | 100 | 9 | 100 | 23 | 100 | 22 | 100 | 42 | 100 | 31 | 100 |
|  | **N (%)** | **Mean^a^** | **SD** | **Mean** | **SD** | **Mean** | **SD** | **Mean** | **SD** | **Mean** | **SD** | **Mean** | **SD** |
| **Gender** |  |  |  |  |  |  |  |  |  |  |  |  |  |
| Male | 43 (56) | 65.0 | 20.5 | 51.9 | 20.8 | 70.6 | 22.9 | 63.1 | 23.7 | 79.4 | 17.3 | 78.6 | 19.2 |
| Female | 34 (44) | 72.3 | 18.9 | 52.5 | 17.7 | 73.9 | 21.2 | 62.4 | 22.2 | 71.0 | 16.6 | 71.0 | 18.1 |
| **Age at diagnosis** *(median, range)* | *6 (0 – 16)* |  |  |  |  |  |  |  |  |  |  |  |  |
| 0 - 5y | 43 (56) | 69.8 | 20.5 | 51.0 | 17.6 | 73.7 | 21.1 | 56.5 | 20.8 | 76.3 | 17.6 | 77.1 | 18.0 |
| 6 - 9y | 22 (29) | 64.1 | 19.4 | 51.9 | 20.6 | 71.5 | 23.9 | 66.8 | 26.5 | 73.9 | 16.6 | 73.5 | 19.1 |
| ≥ 10y | 11 (14) | 70.9 | 20.3 | 57.7 | 25.2 | 68.0 | 24.3 | 70.9 | 20.3 | 75.7 | 20.1 | 69.2 | 22.2 |
| **Attained age** *(median, range)* | *16 (8 – 43)* |  |  |  |  |  |  |  |  |  |  |  |  |
| 8 - 12y | 21 (27) | 70.1 | 24.0 | 53.0 | 16.7 | **83.7^d^** | 20.7 | 59.6 | 4.5 | 71.0 | 16.9 | 73.1 | 20.2 |
| 13 - 17y | 23 (30) | 66.4 | 18.4 | 49.0 | 20.9 | 69.0 | 22.7 | 63.6 | 22.8 | 77.0 | 16.8 | 71.7 | 16.5 |
| ≥ 18y | 33 (43) | 68.2 | 18.8 | 53.8 | 20.4 | 67.2 | 20.4 | 62.7 | 24.7 | 77.8 | 18.1 | 79.0 | 19.7 |
| **Follow-up duration**  *(median, range)* | *10 (2 – 42)* |  |  |  |  |  |  |  |  |  |  |  |  |
| 2 - 5y | 19 (25) | 65.4 | 25.1 | 52.8 | 17.8 | 75.4 | 27.3 | 67.1 | 21.2 | 75.6 | 15.2 | 68.6 | 18.9 |
| 6 - 9y | 21 (27) | 72.0 | 18.0 | 52.1 | 24.8 | 77.6 | 20.9 | 67.7 | 25.8 | 71.0 | 19.8 | 73.1 | 20.1 |
| ≥ 10y | 37 (48) | 67.5 | 18.4 | 51.9 | 17.2 | 67.3 | 19.3 | 60.0 | 22.4 | 78.4 | 16.9 | 79.8 | 17.6 |
| **Site^c^** |  |  |  |  |  |  |  |  |  |  |  |  |  |
| PM | 47 (61) | 68.5 | 19.5 | 49.5 | 19.0 | 67.0 | 22.0 | 56.9 | 20.9 | 76.2 | 18.5 | 73.5 | 20.6 |
| NPM | 11 (14) | 65.3 | 15.4 | 56.6 | 16.4 | 72.3 | 19.8 | 62.0 | 25.2 | 75.1 | 15.2 | 71.8 | 15.8 |
| orbit | 19 (25) | 69.1 | 24.0 | 56.3 | 21.7 | **85.1^e^** | 18.8 | **79.8^f^** | 18.9 | 74.9 | 16.6 | 81.3 | 15.5 |
| **Side** |  |  |  |  |  |  |  |  |  |  |  |  |  |
| Lateral | 63 (82) | 68.6 | 21.1 | 52.2 | 19.9 | 72.4 | 21.9 | 63.7 | 22.4 | 75.1 | 17.8 | 74.2 | 19.2 |
| Midline | 12 (16) | 68.7 | 14.0 | 54.1 | 17.3 | 74.0 | 23.3 | 57.8 | 28.6 | 76.2 | 16.1 | 76.2 | 16.8 |
| **Local treatment** |  |  |  |  |  |  |  |  |  |  |  |  |  |
| RT | 32 (42) | 65.4 | 16.0 | 52.5 | 20.9 | 69.6 | 22.2 | 56.9 | 23.2 | 71.4 | 16.6 | 71.3 | 17.2 |
| Proton | 22 (29) | 73.6 | 20.4 | 51.1 | 18.7 | 80.6 | 23.1 | 66.6 | 23.5 | 74.8 | 20.0 | 71.8 | 21.2 |
| AMORE | 18 (23) | 69.9 | 26.5 | 57.7 | 16.6 | 71.7 | 17.5 | 70.1 | 21.4 | 83.5 | 13.7 | **88.2^h^** | 12.7 |
| Paris-method | 5 (6) | 55.8 | 4.0 | 35.0 | 16.9 | **51.6^g^** | 17.6 | 61.0 | 23.6 | 79.0 | 16.9 | 68.4 | 22.3 |

^a^Mean Rasch transformed scores on scale 0-100; higher scores reflecting better outcome

^b^Only fulfilled by survivors aged ≥12 years

^c^PM: parameningeal site; NPM: head and neck non parameningeal site; orbit: orbital site

^d^Survivors aged 8-12y scored statistically significantly higher on the lips scale compared to the older age groups: compared to 13‑17y (*d* 0.7, p=0.033) and compared to ≥18y (*d* 0.8, p=0.006)

^e^Survivors with orbit site scored significantly higher on the lips scale compared to PM site (*d* 0.9, p=0.002)

^f^Survivors with orbit site scored significantly higher on the jaws scale compared to PM site (*d* 1.1, p=0.000)

^g^Survivors treated according to the Paris-method scored significantly lower on the lips scale compared to survivors treated with protons (*d* -1.3, p = 0.015) or AMORE (*d* -1.2, p = 0.036)

^h^Survivors treated according to the AMORE strategy scores significantly higher on the speech function scale compared to survivors treated with RT (*d* 1.1, 0.001), protons (*d* 0.9, 0.005) or the Paris-method (*d* 1.3, 0.016)

Supplemental table 3:

Correlations between the scale scores across the 3 domains of the FACE-Q

|  | | | **Face** | **Nose** | **Lips** | **Teeth** | **Jaw** | **Psychologic** | **School** | **Social** | **Speech distress** |
| --- | --- | --- | --- | --- | --- | --- | --- | --- | --- | --- | --- |
| **Domains** | **Appearance** | **Face** | X |  |  |  |  |  |  |  |  |
|  |  | **Nose** | **0.56**** | X |  |  |  |  |  |  |  |
|  |  | **Lips** | **0.61**** | **0.60**** | X |  |  |  |  |  |  |
|  |  | **Teeth** | 0.34* | 0.36** | 0.44** | X |  |  |  |  |  |
|  |  | **Jaw** | 0.43** | 0.31* | 0.47** | 0.40* | X |  |  |  |  |
|  | **HRQOL** | **Psychologic** | **0.69**** | **0.51**** | **0.62**** | 0.19 | 0.34* | X |  |  |  |
|  |  | **School** | 0.46* | 0.41** | **0.50**** | 0.07 | 0.34 | **0.66**** | X |  |  |
|  |  | **Social** | **0.55**** | 0.28* | 0.41** | 0.19 | 0.29* | **0.68**** | **0.88**** | X |  |
|  |  | **Speech distress** | 0.08 | -0.06 | 0.004 | 0.10 | 0.23 | 0.15 | 0.31* | 0.24* | X |
|  | **Function** | **Speech function** | 0.20 | 0.004 | 0.11 | -0.01 | 0.22 | 0.22 | 0.41* | 0.31* | **0.75**** |

Pearson correlation coefficients of large (≥0.5) size are presented in bold.

*p <0.05, ** p<0.001

Supplemental figure 2

Number of different adverse events (AEs) versus the scores on the PRO scales (A) psychological functioning (B) and social functioning. Horizontal line corresponds to the cohort mean score on the PRO scales.


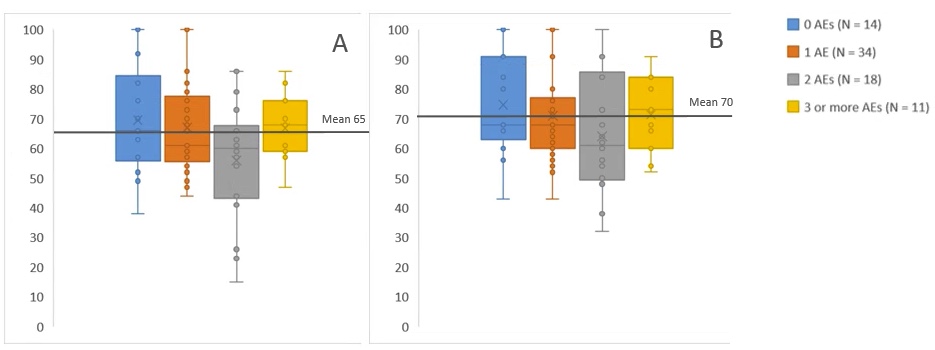


Supplemental table 4: patient characteristics per local treatment strategy

|  | **All**  **N = 77** | **RT**  **N = 32** | **Proton**  **N = 22** | **AMORE**  **N = 18** | **Paris-method**  **N = 5** |
| --- | --- | --- | --- | --- | --- |
| **Gender, male N (%)** | 43 (56) | 21 (66) | 10 (46) | 11 (61) | 5 (80) |
| **Age at diagnosis***,* **y**  *Median (min - max)* | 6 (0 – 16) | 5 (0 – 16) | 5 (1 – 12) | 7 (2 – 13) | 8 (5 – 15) |
| **Attained age, y**  *Median (min - max)* | 16 (8 – 39) | 19 (10 – 39) | **12 (8 – 22)^a^** | 21 (8 – 34) | **13 (12 – 19)^b^** |
| **Follow-up duration, y**  *Median (min - max)* | 10 (2 – 30) | 14 (3 – 30) | **7 (3 – 14)^c^** | 12 (3 – 27) | **6 (2 – 8)^d^** |
| **Site, N (%)** |  |  |  |  |  |
| PM | 47 (61) | 21 (66) | 14 (64) | 7 (39) | **5 (100)^e^** |
| NPM | 11 (14) | 3 (9) | 3 (14) | 5 (28) | 0 |
| orbit | 19 (25) | 8 (25) | 5 (23) | 6 (33) | 0 |
| **Side, N (%)** |  |  |  |  |  |
| Lateral | 63 (84) | 25 (81) | 16 (76) | 17 (94) | 5 (100) |
| Midline | 12 (16) | 6 (19) | 5 (24) | 1 (6) | 0 |
| **Country of residence, N (%)** |  |  |  |  |  |
| United Kingdom | 31 (40) | 20 (63) | 11 (50) | 0 | 0 |
| United States | 6 (8) | 0 | 6 (27) | 0 | 0 |
| France | 8 (10) | 2 (6) | 1 (5) | 0 | 5 (100) |
| The Netherlands | 32 (42) | 10 (31) | 4 (18) | 18 (100) | 0 |

Kruskal-Wallis test for comparison of medians. Fisher exact test for comparison of proportions. Statistically significant differences are bold.

^a^Attained age in proton-treated group significantly younger compared to RT (p = 0.000) and AMORE (p = 0.000).

^b^Attained age in Paris-method group significantly younger compared to RT (p = 0.000) and AMORE (p = 0.000)

^c^Follow-up period in proton-treated group significantly shorter compared to RT (p = 0.000) and AMORE (p = 0.000).

^d^Follow-up period in Paris-method group significantly shorter compared to RT (p = 0.000) and AMORE (p = 0.000)

^e^Significantly higher proportion of PM site tumors in Paris-method group compared to AMORE treated group (p = 0.037).
