# Supplementary material for: Patient‐reported outcomes in childhood head and neck rhabdomyosarcoma survivors and their relation to physician‐graded adverse events—A multicenter study using the FACE‐Q Craniofacial module
Source: Cancer Med. 2022 Oct 7;12(4):4739–50. doi: 10.1002/cam4.5252 (PMC9972026; doi:10.1002/cam4.5252)
Supplement: Supplementary file 2 — Appendix S1 [file CAM4-12-4739-s002.docx]

Supplemental data B

Definition of Adverse Events, selection of Common Terminology Criteria for adverse events version 4.0. Conditions are classified as mild (grade 1), moderate (grade 2), severe (grade 3), life-threatening or disabling (grade 4) (grade 5 (fatal) not included in the current study).

1. Musculoskeletal deformity

|  | **Grade** | | | | |
| --- | --- | --- | --- | --- | --- |
| **Adverse event** | **0** | **1** | **2** | **3** | **4** |
| **Musculoskeletal deformity** | - | Cosmetically and functionally insignificant hypoplasia | Deformity, hypoplasia, or asymmetry able to be covered | -Significant deformity, hypoplasia or asymmetry, not covered  -Disabling | Orbital exenteration |

1. Speech abnormality

|  | **Grade** | | | | |
| --- | --- | --- | --- | --- | --- |
| **Adverse event** | **0** | **1** | **2** | **3** | **4** |
| **Rhinolalia aperta (nasal aspirate sound)†** | **-** | **Mild change of speech, no effect on audibility** | **Moderate change of speech, influences audibility** | **Barely understandable, verbal communication limited** | **-** |
| **Dysarthria/**  **voice alteration** | **-** | **-Mild slurred speech**  **-Mild or intermittent change from normal voice** | **-Moderate impairment of articulation or slurred speech**  **-Moderate or persistent change from normal voice; still understandable** | **-Severe impairment of articulation or slurred speech**  **-Severe voice changes including predominantly whispered speech**  **-May require frequent repetition or face-to-face contact for understandability**  **-May require assistive technology** | **-** |

1. Oral malfunction

|  | **Grade** | | | | |
| --- | --- | --- | --- | --- | --- |
| **Adverse event** | **0** | **1** | **2** | **3** | **4** |
| **Taste alteration* (Dysgeusia)** | **-** | **Altered taste** | **-Changed diet**  **-Noxious, unpleasant**  **-Loss of taste** | **-** | **-** |
| **Trismus** | **-** | **Decreased range of motion (ROM)** | **Decreased ROM, requiring small bites, soft foods or purees** | **Decreased ROM, inability to adequately aliment or hydrate orally** | **-** |
| **Xerostomia**  **(dry mouth)** | **-** | **Symptomatic (e.g. dry or thick saliva) without significant dietary alterations** | **-Moderate symptoms**  **-Oral intake alterations** | **-Inability to adequately aliment orally**  **-TPN/tube feeding indicated** | **-** |

1. Hearing impairment

|  | **Grade** | | | | |
| --- | --- | --- | --- | --- | --- |
| **Adverse event** | **0** | **1** | **2** | **3** | **4** |
| **Hearing***  **(subjective)** | **-** | **-** | **Hearing loss** | **Hearing loss requiring intervention** | **Profound bilateral hearing loss (>90dB)** |

*Not available in CTCAEv4.0, description as defined in CTCAEv3.0

1. Ocular problem

|  | **Grade** | | | | |
| --- | --- | --- | --- | --- | --- |
| **Adverse event** | **0** | **1** | **2** | **3** | **4** |
| **Eyelid function disorder** | **-** | **-Asymptomatic** | **-Symptomatic**  **-Non-operative intervention indicated**  **-Limiting instrumental ADL**** | **-Limiting self care ADL****  **-Operative intervention indicated** | **-** |
| **Diplopia*** | **-** | **Intermittently symptomatic, intervention not indicated** | **Symptomatic and interfering with function but not interfering with ADL** | **-Symptomatic and interfering with ADL**  **-Surgical intervention indicated** | **Disabling** |
| **Enophtalmus*** | **-** | **Asymptomatic** | **-Symptomatic**  **-Limiting instrumental ADL**** | **-Limiting self care ADL****  **-Disabling** | **-** |
| **Exophtalmus†** | **-** | **Asymptomatic** | **-Symptomatic**  **-Limiting instrumental ADL**** | **-Limiting self care ADL****  **-Disabling** | **-** |
| **Strabismus** | **-** | **Asymptomatic** | **-Symptomatic**  **-Limiting instrumental ADL**** | **-Limiting self care ADL****  **-Disabling** | **-** |
| **Dry eye** | **-** | **-Asymptomatic**  **-Mild symptoms relieved by lubricants** | **-Symptomatic**  **-Multiple agents indicated**  **-Limiting instrumental ADL**** | **-Decrease in visual acuity (<20/40)**  **-Limiting self care ADL**** | **-** |
| **Watering eyes** | **-** | **Symptomatic** | **Intervention indicated** | **Operative intervention indicated** | **-** |
| **Blurred vision** | **-** | **Symptomatic** | **Limiting instrumental ADL**** | **Limiting self care ADL**** | **-** |
| **Cataract** | **-** | **Asymptomatic** | **-Symptomatic: moderate decrease visual acuity (20/40 or better)** | **-Marked decrease visual acuity (20/40-20/200)**  **-Operative intervention indicated** | **-Blindness (20/200 or worse) in affected eye** |
| **Conjunctivitis** | **-** | **-Asymptomatic**  **-Intervention not indicated** | **-Symptomatic**  **-Limiting instrumental ADL****  **- Topical intervention indicated** | **Limiting self care ADL**** | **-** |
| **Flashing lights/ floaters** | **-** | **Symptomatic** | **Limiting instrumental ADL**** | **Limiting self care ADL**** |  |
| **Glaucoma** | **-** | **-Elevated intraocular pressure with single topical agent**  **-No visual field deficit** | **-Early visual field deficit**  **-Multiple agents indicated (oral/topical)**  **-Limiting instrumental ADL**** | **-Marked visual field deficits**  **-Operative intervention indicated**  **-Limiting self care ADL**** | **-Blindness (20/200 or worse)** |
| **Keratitis**  **(corneal inflammation, ulceration)** | **-** | **-** | **-Symptomatic**  **-Medical intervention indicated**  **-Limiting instrumental ADL**** | **-Limiting self care ADL****  **-Decline in vision 20/40-20/200** | **Perforation or blindness (20/200 or worse)** |
| **Optic nerve disorder** | **-** | **Asymptomatic** | **Limiting vision of the effected eye (20/40 or better)** | **-Limiting vision of the affected eye (20/40-20/200)** | **Blindness (20/200 or worse)** |
| **Papilledema** | **-** | **-Asymptomatic**  **-No visual field defects** | **-Symptomatic decline in vision**  **-Visual field defect present sparing the central 20 degrees** | **-Marked visual field defect (20/40-20/200)** | **Blindness (20/200 or worse)** |
| **Photophobia** | **-** | **Symptomatic** | **Limiting instrumental ADL**** | **Limiting self care ADL**** | **-** |
| **Retinal detachment** | **-** | **Asymptomatic** | **Exsudative and visual acuity (20/40 or better)** | **-Rhegmatogenous or exsudative detachment**  **-Operative intervention indicated**  **-Decline in vision (20/40-20/200)** | **Blindness (20/200 or worse)** |
| **Retinal vascular disorder** | **-** | **-** | **Topical medication indicated** | **-Intravitreal medication**  **-Operative intervention indicated** | **-** |

*Not available in CTCAEv4.0, description as defined in CTCAEv3.0

† item added based on results from pilot study

**Activities of Daily Living (ADL) Instrumental ADL refer to preparing meals, shopping for groceries or clothes, using the telephone, managing money etc. Self care ADL refer to bathing, (un)dressing, feeding self, using the toilet, taking medications and not bedridden.
